# Bacterial and archaeal communities in the deep-sea sediments of inactive hydrothermal vents in the Southwest India Ridge

**DOI:** 10.1038/srep25982

**Published:** 2016-05-12

**Authors:** Likui Zhang, Manyu Kang, Jiajun Xu, Jian Xu, Yinjie Shuai, Xiaojian Zhou, Zhihui Yang, Kesen Ma

**Affiliations:** 1 Marine Science & Technology Institute Department of Environmental Science and Engineering, Yangzhou University, No. 196 Huayang West Road, Hanjiang District Yangzhou City, Jiangsu Province, 225127, China; 2 College of Plant Protection, Agricultural University of Hebei, Baoding City, Hebei Province 071001, China; 3 Department of Biology, University of Waterloo, Waterloo, Ontario, Canada

## Abstract

Active deep-sea hydrothermal vents harbor abundant thermophilic and hyperthermophilic microorganisms. However, microbial communities in inactive hydrothermal vents have not been well documented. Here, we investigated bacterial and archaeal communities in the two deep-sea sediments (named as TVG4 and TVG11) collected from inactive hydrothermal vents in the Southwest India Ridge using the high-throughput sequencing technology of Illumina MiSeq2500 platform. Based on the V4 region of 16S rRNA gene, sequence analysis showed that bacterial communities in the two samples were dominated by *Proteobacteria*, followed by *Bacteroidetes*, *Actinobacteria* and *Firmicutes*. Furthermore, archaeal communities in the two samples were dominated by *Thaumarchaeota* and *Euryarchaeota*. Comparative analysis showed that (i) TVG4 displayed the higher bacterial richness and lower archaeal richness than TVG11; (ii) the two samples had more divergence in archaeal communities than bacterial communities. Bacteria and archaea that are potentially associated with nitrogen, sulfur metal and methane cycling were detected in the two samples. Overall, we first provided a comparative picture of bacterial and archaeal communities and revealed their potentially ecological roles in the deep-sea environments of inactive hydrothermal vents in the Southwest Indian Ridge, augmenting microbial communities in inactive hydrothermal vents.

Deep-sea hydrothermal vent environments exhibit complex dynamic habitats that are characterized with steep thermal and chemical gradients^1^. Microorganisms thriving in hydrothermal vent environments have been extensively studied by cultivation-dependent and -independent approaches^2^. From these environments, thermophilic archaeal members of the orders *Thermococcales*, *Methanococcales*, *Archaeoglobales*, and *Aquificales*^3^,^4^,^5^, and mesophilic and thermophilic bacterial members of *Epsilon-proteobacteria*^6^,^7^ have been detected. However, active hydrothermal vents eventually become inactive ones, which may last for thousands of years^8^. Inactive hydrothermal vents provide potential habitats for microbial communities. Many reports have shown that inactive hydrothermal vents harbor a completely different assemblage of microorganisms, compared to active hydrothermal vents. For example, *Thaumarchaeota*, which are usually not major archaea in active vents, have been found to be widespread not only in several inactive hydrothermal vents^8^,^9^,^10^, but also in diffuse flow chimneys^11^. In addition, *Alpha-, Beta-, Delta*-, and *Gamma-proteobacteria* are dominant bacteria in several inactive hydrothermal vents^8^,^10^,^12^.

The Southwest Indian Ridge is a boundary between the Antarctic and African plates and extends from the Bouvet triple junction in the Atlantic Ocean to the Rodriguez triple junction in the Indian Ocean^13^. The first active hydrothermal vent (E 49°39, S 37°47) in the Southwest Indian Ridge was discovered in 2011, which is characterized with low temperatures and enrichment for chimney sulfides, such as pyrite, marcasite, sphalerite, and chalcopyrite^14^. In previous studies, arsenite-resistant bacteria were isolated from deep-sea sediments in the Southwest Indian Ridge^15^, and microbial sulfur cycle in two hydrothermal chimneys on the Southwest Indian Ridge were also revealed^16^. Furthermore, microbial communities in semi-consolidated carbonate sediments (51.009^o^E, 37.6081^o^S) of the Southwest Indian Ridge were investigated by traditional 16S rDNA clone library analysis^9^; however, extensive microbial diversity and abundance in inactive hydrothermal vents in the Southwest Indian Ridge have not been well understood.

The primary goal of this study was to reveal microbial communities of two deep-sea sediments located at different regions in inactive hydrothermal vents in the Southwest Indian Ridge. For this purpose, the V4 region of 16S rRNA gene were sequenced via an Illumina MiSeq 2500 platform, which is a high-throughput sequencing technology that has been frequently used to investigate microbial community structure in various environments^17^,^18^,^19^,^20^,^21^. In this study, bacteria and archaea covering 15 phyla, 34 classes, 58 orders, 101 families and 141 genera were detected, augmenting the microbial community structure in inactive hydrothermal vents in the Southwest Indian Ridge. Furthermore, our results revealed that bacterial communities were dominated by *Proteobacteria* and *Bacteroidetes* and archaeal communities were mostly composed of *Thaumarchaeota* and *Euryarchaeota.* To the best of our knowledge, this is the first report on bacterial and archaeal communities of the deep-sea sediments in inactive hydrothermal vents in the Southwest Indian Ridge by Illumina high-throughput sequencing.

## Results

### Mineralogical and geochemical characteristics of the samples

Mineralogical and geochemical characteristics of the two samples were determined using X-ray diffraction phase, scanning electron microscope, carbon and oxygen isotope, and trace element analyses^22^. Generally, the two samples had similar mineralogical and geochemical characteristics summarized in Table 1. The total content of Rare Earth Element in the two samples tended to be low with a range of 19.82 ~ 21.08 × 10^−6^. The two samples had abundant trace elements, such as Sr and Ba, but depleted in siderophile elements. Major calcite (>95%) and lower content of clay minerals, aragonite and quartz were present in the two samples. Furthermore, the two samples had the higher content of δ^18^O_PDB_ than δ^13^C_PDB_. However, the TVG4 sample was white sticky mud while the TVG11 sample was black loose mud and sand, which may lead to different microbial community structure.

### Sequencing data

Sequencing information, diversity index, and estimators of richness were summarized in Table 2. The Illumina-based analysis of the hypervariable V4 region of 16S rRNA gene produced 78,461 and 358,589 total tags for bacteria and archaea, respectively. After filtering and removing potential erroneous sequences, a total of 74,980 and 311,035 effective tags were obtained for bacteria and archaea, respectively.

**Figure 1:**
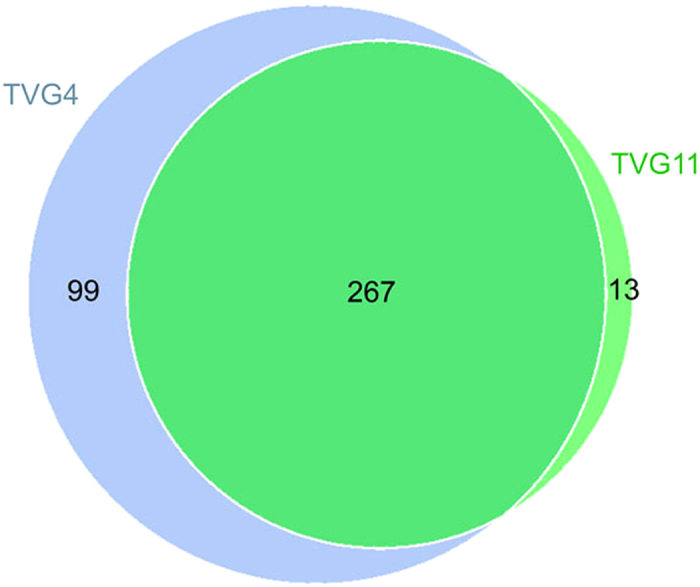
Venn diagrams of the OTUs for bacterial diversity. Unique and shared OTUs between the two samples were based on 97% similarity. The numbers inside the diagram are the numbers of OTUs.

Based on 97% similarity (Table 2), a total of 366 and 280 OTUs (operational taxonomic units) for bacterial diversity were obtained in TVG4 and TVG11 respectively, and a total of common 19 OTUs for archaeal diversity were obtained in TVG4 and TVG11. The Venn diagram for bacterial diversity showed that TVG4 and TVG11 shared 267 OTUs, 99 and 13 OTUs were unique in TVG4 and TVG11 respectively (Fig. 1). Interestingly, no unique archaeal OTU was observed in TVG4 and TVG11, besides the shared 19 OTUs.

**Figure 2:**
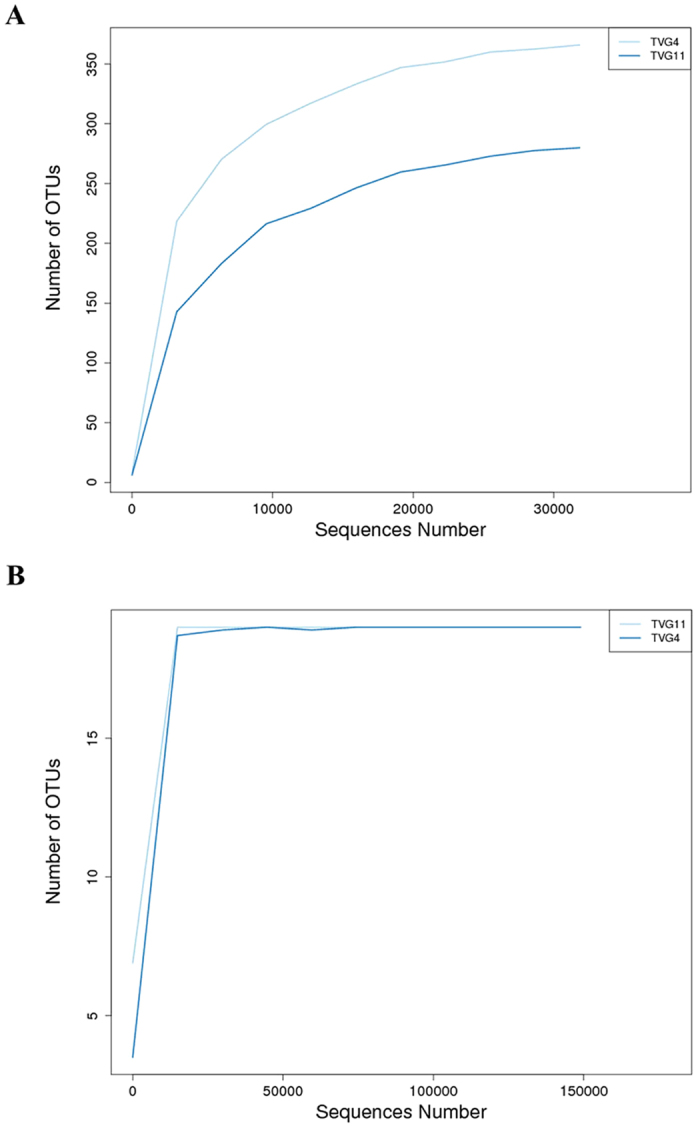
Rarefaction curves of 16S rDNA sequences for bacterial diversity (**A**) and archaeal diversity (**B**) in the two samples. Curves were calculated based on OTUs at 97% similarity.

In alpha diversity analysis, rarefaction curves, Chao1 index and Shannon index were generated based on 97% similarity. Rarefaction curve analysis of OTUs in the two samples indicated that TVG4 had more bacterial species than TVG11 (Fig. 2A), and the two samples had the same archaeal species (Fig. 2B). This was confirmed by Chao1 index (Table 2). Furthermore, both rarefaction curves for bacterial and archaeal species approached an asymptote (Fig. 2), suggesting that the sampling depths were sufficient to capture overall microbial diversities in the two samples. In addition, Shannon’s diversity index showed that the order of the diversity, ranging from high to low, was TVG4 for bacterial diversity (Shannon = 5.76), TVG11 for bacterial diversity (Shannon = 3.99), TVG11 for archaeal diversity (Shannon = 3.05), and TVG4 for archaeal diversity (Shannon = 2.05) (Table 2).

### Microbial community analysis

A total of 386,015 effective tags were obtained, including 74,980 bacterial tags and 311,035 archaeal tags (Table 2). Bacterial tags covered 13 phyla, 32 classes, 56 orders, 97 families, 138 genera, and archaeal tags covered 2 phyla, 2 classes, 2 orders, 2 families, and 3 genera. Bacterial and archaeal sequences from the two samples were further analyzed at the phylum, class and genus levels.

**Figure 3:**
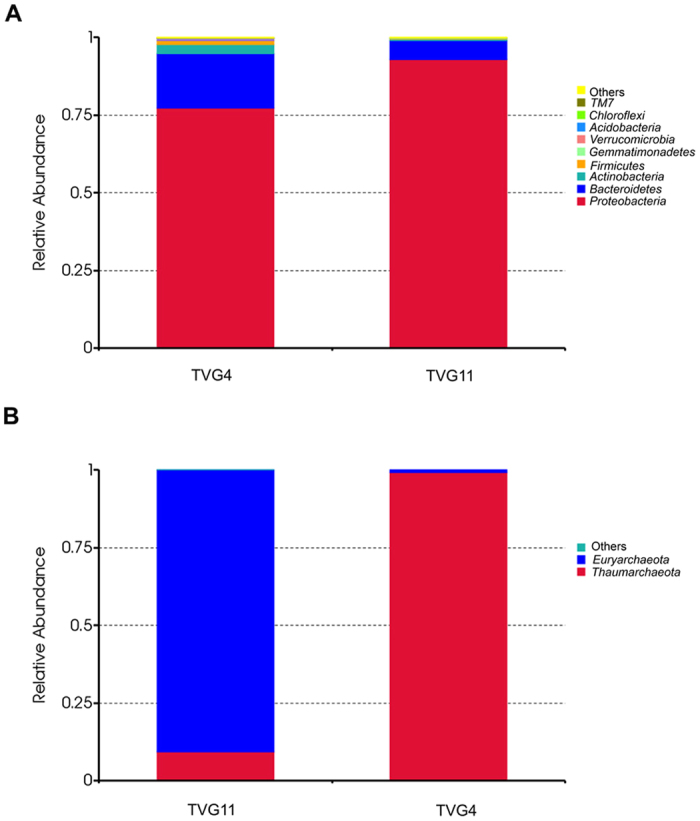
Relative abundance of bacteria (**A**) and archaea (**B**) at the phylum level. Each color represents the percentage of the phylum in the total effective tags of each sample.

Based on average abundance analysis, at the phylum level, *Proteobacteria* (77.4%), *Bacteroidetes* (17.5%), *Actinobacteria* (2.9%) and *Firmicutes* (1.3%) were the four major phyla of bacteria in TVG4 (Fig. 3A). *Proteobacteria* (92.9%) and *Bacteroidetes* (6.0%) were the two major phyla of bacteria in TVG11 (Fig. 3A). In addition, *Gemmatimonadetes*, *Verrucomicrobia*, *Acidobacteria*, *Chloroflexi*, TM7 and other bacteria were found in TVG4 and TVG11 with low abundance (<1%) (Fig. 3A). While TVG4 had lower abundance of *Proteobacteria* than TVG11, TVG4 had higher abundance of *Bacteroidetes*, *Actinobacteria* and *Firmicutes* than TVG11. Thus, the results suggest that *Proteobacteria* and *Bacteroidetes* are dominant phyla in TVG4 and TVG11.

At the phylum level, 99.57% of sequences belonged to *Euryarchaeota* phylum while only 0.43% of sequences were members of *Thaumarchaeota* phylum in TVG4 (Fig. 3B), suggesting that *Euryarchaeota* are major phyla in TVG4. In contrast, 88.22% of sequences belonged to *Thaumarchaeota* phylum while 11.78% of sequences were members of *Euryarchaeota* in TVG11 (Fig. 3B), indicating that *Thaumarchaeota* are predominant phyla in TVG11. Thus, the results suggest the significant divergence in the relative abundance of archaeal phyla between TVG11 and TVG4, although no unique OTU was observed for them.

**Figure 4:**
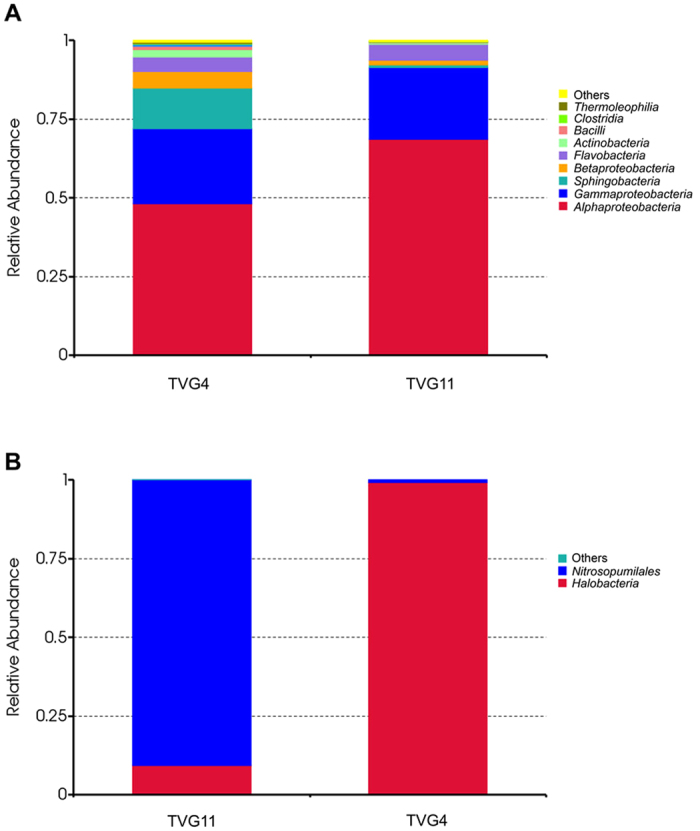
Relative abundance of bacteria (**A**) and archaea (**B**) at the class level. Each color represents the percentage of the class in the total effective tags of each sample.

Of the 32 classes of bacteria, 28 classes were found in TVG4 and TVG11. Based on average relative abundance, *Alpha-proteobacteria* (48.2% for TVG4 and 68.7% for TVG11), *Gamma-proteobacteria* (23.7% for TVG4 and 22.7% for TVG11), *Sphingobacteria* (12.8% for TVG4 and 1.0% for TVG11), *Beta-proteobacteria* (5.32% for TVG4 and 1.54% for TVG11), *Flavobacteria* (4.63% for TVG4 and 5.08% for TVG11) were the five major classes of bacteria in the two samples (Fig. 4A). In addition, *Actinobacteria*, *Bacilli*, *Clostridia*, *Thermoleophilia* and others were found with low abundance in the two samples (Fig. 4A). Thus, *Alpha-proteobacteria* was the major class in the two samples, followed by *Gamma-proteobacteria*, *Sphingobacteria, Beta-proteobacteria* and *Flavobacteria*.

*Halobacteriales* of *Euryarchaeota* phylum and *Nitrosopumilales* of *Thaumarchaeota* phylum were the main classes of archaea in the two samples (Fig. 4B). While TVG11 had a low relative abundance of *Halobacteriales* (9.3%) and a high relative abundance of *Nitrosopumilales* (90.7%), TVG4 had a high relative abundance of *Halobacteriales* (99.3%) and a low relative abundance of *Nitrosopumilales* (0.7%). Overall, the two samples had same archaeal species, but different relative abundance.

**Figure 5:**
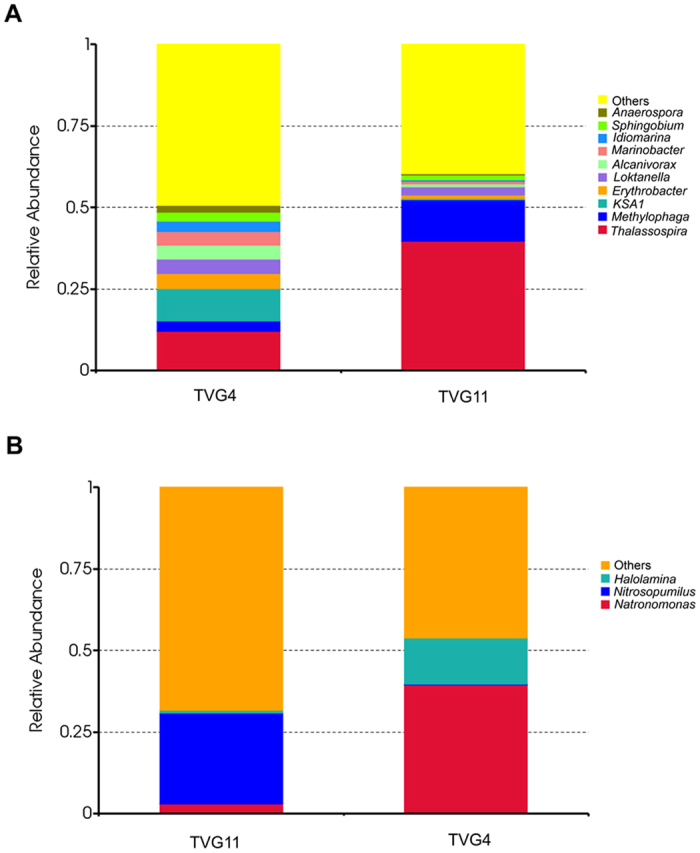
Relative abundance of bacteria (**A**) and archaea (**B**) at the genus level. Each color represents the percentage of the genus in the total effective tags of each sample.

At the genus level, the most abundant bacterial genus in the two samples was *Thalassospira* with a relative abundance of 25.8%; other nine abundant genera were *Methylophaga*, *KSA1*, *Erythrobacter*, *Loktanella*, *Marinobacter*, *Alcanivorax*, *Idiomarina*, *Sphingobium* and *Anaerospora* (Fig. 5A). On the other hand, at the genus level, the most abundant archaeal genus in TVG4 was *Natronomonas* (39.5%), followed by *Halolamina* (14.2%), while *Nitrosopumilus* was the most abundant archaeal genus in TVG11 with a relative abundance of (27.9%) (Fig. 5B).

### Beta diversity index analysis of the samples

**Figure 6:**
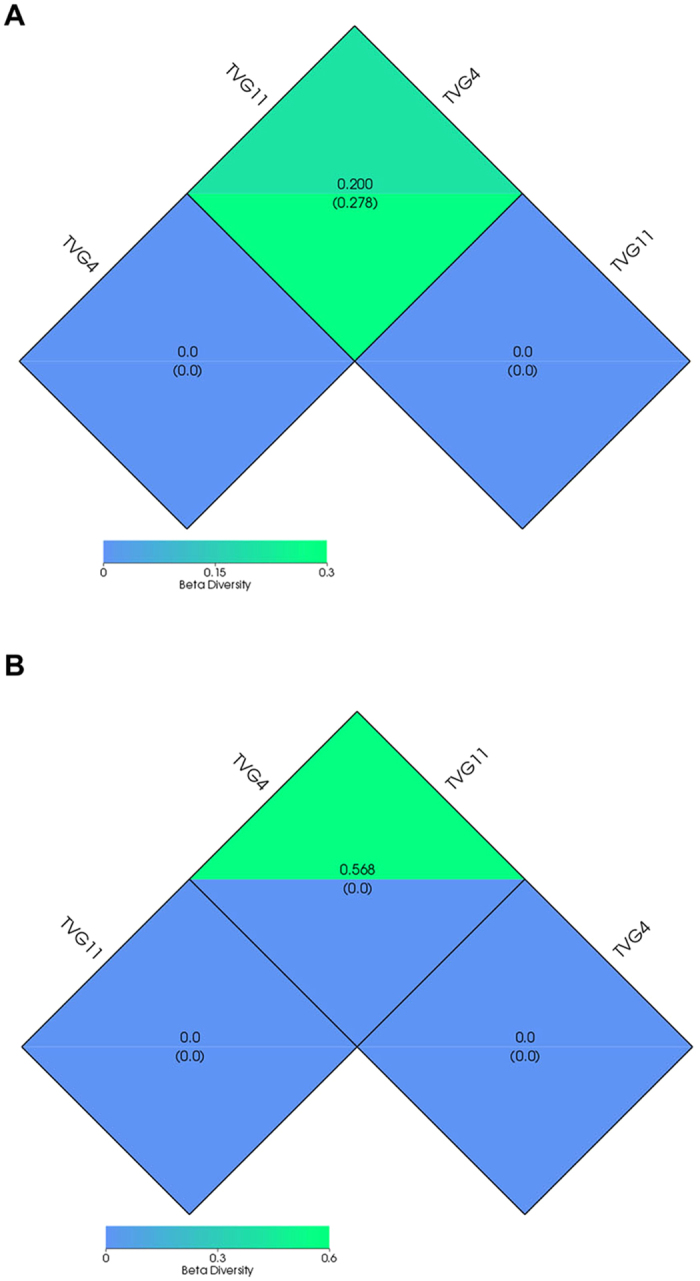
Beta diversity index of the bacterial and archaeal communities in the two samples. Beta diversity index were measured based on weighted Unifrac and unweighted Unifrac distances. The upper and under numbers in the grid represents the weighted Unifrac and unweighted Unifrac distances, respectively. (**A**). Beta diversity index for bacterial communities; (**B**). Beta diversity index for archaeal communities.

Based on the weighted UniFrac distance and unweighted UniFrac distance cluster analysis, a dissimilarity coefficient for the two samples was measured to estimate the divergence of microbial species between them. The lower dissimilarity coefficients suggest the less divergence of microbial species. In this study, the dissimilarity coefficients for the two samples were measured to be 0.2 and 0.568 for bacterial and archaeal diversities, respectively (Fig. 6), suggesting that more divergence in the two samples was observed for archaeal species than for bacterial species.

## Discussion

High-throughput sequencing techniques provide powerful tools for studying microbial communities in extreme environments, including deep-sea environments. For example, MiSeq sequencing platform PE250 or PE300 is capable of reading long DNA fragments and is widely used for microbial 16S rDNA sequencing^23^, allowing us to detect rare microorganisms with low relative abundance (<0.01%) that would been masked by dominant populations if techniques with lower resolution had been applied^24^. Thus, the accuracy of species abundance will be greatly enhanced by MiSeq sequencing platform. Currently, Miseq sequencing platform has been a prior method to study microbial diversities in various environments. To discern more fully bacterial and archaeal communities in deep-sea sediments from inactive hydrothermal vents at the Southwest Indian Ridge, the V4 region of 16S rRNA gene from the two sample DNA were amplified and phylogenetically analyzed by a barcoded IIIumina high-throughput sequencing.

The phylum *Proteobacteria* was dominant in the taxonomic groups of the two samples in this study. Within this phylum, *Alpha-proteobacteria*, *Gamma-proteobacteria*, and *Sphingobacteriia*, were the three main classes, which are similar to the reports on the deep-sea sediments from Iheya North and Iheya Ridge^19^ and metal-rich vent deposits from Pacific Ocean hydrothermal fields^25^,^26^. In the Southwest Indian ridge, similar observations were also made in semi-consolidated carbonate sediment samples^9^. Previous studies have shown that *Epsilon-proteobacteria* are dominant bacteria identified from active hydrothermal vents, and play a role in carbon and sulfur cycles^6^,^12^,^27^,^28^. However, *Epsilon-proteobacteria* was not detected in the two samples in this study. Compared to bacterial communities in active vents, which are dominated by *Epsilon-proteobacteria*, bacterial communities in inactive vents are mostly composed of *Alpha*-, *Beta*-, *Delta*-, and *Gamma-proteobacteria*^8^,^10^,^12^. Our results augment the hypothesis that while *Epsilon-proteobacteria* prefers active hydrothermal vents, *Alpha-, Beta-, Delta*-, and *Gamma-proteobacteria* prefer inactive hydrothermal vents.

*Bacteroidetes* was the second most abundant phylum in the two samples examined in this study and contained three classes, i.e. *Sphingobacteria*, *Flavobacteria*, and *Bacteroidia*. Previous studies have shown that *Bacteroidetes* are widely distributed in many inactive and active deep-sea hydrothermal vents^7^,^12^. It was also detected as one of the most abundant phylum in methane seep sediments in the Nankai Trough^29^. In this study, the abundance of *Bacteroidetes* in TVG4 was higher than that in TVG11, likely as a result of environmental difference between these two samples. Other dominant groups of bacteria identified in this study include members of *Gemmatimonadetes*, *Verrucomicrobia*, *Acidobacteria*, *Chloroflexi*, TM7, etc., which are known to be widely distributed in crustal fluids, deep-sea sediments, and inactive hydrothermal chimneys, but rare in active hydrothermal regions^8^,^12^,^25^,^30^.

*Euryarchaeota*, *Crenarchaeota*, and *Thaumarchaeota* are known to be three major archaeal phyla in global environments. In this study, only *Euryarchaeota* and *Thaumarchaeota* were identified. Specifically, *Thaumarchaeota* were the major phylum in TVG11 while *Euryarchaeota* were the predominant archaea in TVG4. Previous studies have shown that *Thaumarchaeota* are widely found not only in several inactive hydrothermal vents from Southwest Indian Ridge, Western Pacific Ocean and Central Indian Ridge^8^,^9^ as well as in diffuse flow chimneys from Arctic Mid-Ocean Ridge^31^. Besides, members of *Thaumarchaeota* are found in marine, freshwater, soils, and anoxic subsurface sediments^32^, all which possess the dissimilatory ammonia oxidation pathway and gain energy by ammonia oxidation^33^. However, few *Thaumarchaeota* has been found in active hydrothermal vents, which harbor a great number of typical (hyper)thermophilic archaea, such as *Desulfurococcales, Thermococcales* and *Archaeoglobales*^4^,^27^,^34^,^35^,^36^. *Thaumarchaeota* also dominated the archaeal communities in the semi-consolidated carbonate deep-sea sediments in the Southwest Indian ridge^9^, which is consistent with our observations. Taken together, these results suggest a wide presence of *Thaumarchaeota* in inactive hydrothermal vents.

Besides *Thaumarchaeota, Natronomonas* and *Halolamina* were dominant *Euryarchaeota* in TVG4 in this work. To the best of our knowledge, this is the first report on the presence of *Natronomonas* and *Halolamina* in inactive hydrothermal vents. Recently, the analysis of microbial diversity in the new deep-sea hypersaline Lake Thetis located in the Western part of the Mediterranean Ridge indicated that neither *Natronomonas* nor *Halolamina* was not detected^37^,^38^. The presence of *Natronomonas* and *Halolamina* in the Southwest Indian ridge may be relevant to the geochemical environments.

The genera *Nitrospira* of the *Nitrospinae* phylum are mainly widespread in marine environments including metal-rich sediments from Green Bay and western Pacific Ocean^25^,^39^, and participate in nitrogen cycling by converting nitrites to nitrates^40^,^41^. These two bacteria were also detected in the two samples. In this study, *Nitrospira* accounted for 0.01% of total bacteria in the two samples in Table 2. Furthermore, *Rhizobiales*, which participates in nitrogen-fixing^42^, accounted for 2.2% of total bacteria in the two samples (Table 3). Besides bacteria, archaea also play an important role in the oxidation of ammonia to nitrite and in N_2_ fixation^32^. For example, *Thaumarchaeota* harbors the ammoniamonooxygenase subunit A (amoA) gene, and the encoded AmoA can oxidize ammonia to nitrite^43^. The genera *Nitrosopumilus* of the phylum *Thaumarchaeota*, an ammonia-oxidizing archaea, was detected with a high relative abundance (27.9%) in TVG11 but a low relative abundance (20%) in TVG4 (Table 3). The presence of these bacterial and archaeal communities that are associated with nitrogen metabolism in this study indicates a likely involvement of these microbes in the process of nitrogen cycle.

Increasing evidences suggest that bacteria play a key role in the formation of deep-sea metallic mineral^44^. For example, *Rhodococcus*, *Caulobacter*, *Hyphomicrobium* and *Acinetobacter* are known to be involved in metal metabolism^45^,^46^,^47^,^48^. In this study, these four genera accounted for 0.59% of the bacteria and had a higher abundance in TVG11 (0.39%) than in TVG4 (0.20%) (Table 3). The genus *Hyphomicrobium* of the *Alpha-proteobacteria* class, which is known to associate with metal metabolism^45^, accounted for 0.32% of the bacteria in this study. *Rhodococcus*, one genus of Mn-oxidizing bacteria, was detected with a relative abundance of 0.023%. *Rhodococcus*, which was first isolated from submarine basalts, can absorb metals such as Co, Cu, Ni and Mn, thus facilitating metal enrichment^46^. The genera *Acinetobacter* of the *Gamma-proteobacteria* class, which is involved in nutrient metal acquisition and metabolism^47^, were detected in the two samples with a 0.13% of relative abundance. Furthermore, *Caulobacter* within the *Alpha-proteobacteria* class can absorb and metabolize a variety of metals including Mn, Co, and Fe^48^. *Caulobacter* was detected in the two samples with a 0.11% of relative abundance. Overall, the presence of metal-utilizing bacteria of the samples in this study suggests a mechanism of environment induced microbial adaptation.

*Alpha*-, *Delta*-, *Gamma*- and *Epsilon-proteobacteria* are known to mediate sulfide reduction and oxidation^49^, which is one of the most important microbial chemosynthetic pathways in deep-sea hydrothermal ecosystems^50^. In this study, potential sulfide oxidation/reduction microbes comprised over 10.977% of the bacteria, including *Alpha*- and *Delta-proteobacteria* (Table 3). The order *Rhodobacteraceae* of *Alpha-proteobacteria* class and the order *Desulfobacterales* of *Delta-proteobacteria* class, which accounted for 10.97% and 0.007% of the bacteria in this study (Table 3), respectively, have been detected in many hydrothermal chimneys with functions of sulfide oxidation or reduction^5^,^25^,^51^.

Besides the bacteria and archaea associated with nitrogen and sulfur cycling and metal metabolism, the genus *Planctomycetes* was detected with a total abundance of 0.08% in this study (Table 3). Chistoserdova *et al.* suggested a possible role for *Planctomycetes* in the evolution of the methane cycle on Earth^52^. Thus, the presence of *Planctomycetes* in the two samples of our study is thought to participate in CH_4_ oxidation.

Alpha diversity analysis showed that bacterial richness in TVG4 were higher than that in TVG11 while archaeal richness in TVG4 were lower than that in TVG11 as revealed by Shannon’s diversity index, suggesting that environmental parameters probably have a significant impact on microbial community. Beta diversity analysis showed that the two samples had more difference in archaeal abundance than bacterial abundance. These results suggest that the distance to active hydrothermal vents and ambient environmental parameters probably have an important influence on the formation of microbial communities, even in the same oceanic ridge.

In conclusion, we revealed for the first time the comparative analysis of the microbial diversity in different sediments of the Southwest Indian Ridge using the high-throughput sequencing technology of Illumina MiSeq 2500 platform. Although the taxonomies of microbial communities in the two samples are largely similar, the abundances of most of the taxa differ between the two samples. Furthermore, our results revealed that bacterial communities were dominant by *Proteobacteria* and *Bacteroidetes* and archaeal communities were mostly composed of *Thaumarchaeota* and *Euryarchaeota.* These bacterial and archaeal communities would be potentially involved in nitrogen and sulfur cycling, and metal metabolism, suggesting that they play the important roles in ecological function in inactive deep-sea hydrothermal vents.

## Methods

### Sample sites and collection

The sediment samples were collected from the inactive hydrothermal vents using a television grab bucket of the R/V Da-Yang-Yi-Hao conducted by the China Ocean Mineral Resource R&D Association in 2009. The positions of the samples were as follows: TVG4 (E 50.9277°, S 37.6251°, ~2086 m) and TVG11 (E 50.9643°, S 37.6174°, ~1985 m). After sample collection, the TVG4 and TVG11 samples were frozen at −20 °C immediately before being processed.

### Mineralogical and geochemical analysis

Mineralogical and geochemical characteristics of the two samples were performed by X-ray diffraction phase, scanning electron microscope, carbon and oxygen isotope, and trace element analyses at China University of Geosciences at Beijing in 2013^22^.

### DNA extraction and PCR amplification

Total microbial community DNA was directly extracted from the two samples by using Mo Bio soil DNA extraction kit (Carlsbad, CA USA) according to manual instructions. 0.25 g (wet weight) of the TVG4 and TVG11 samples was used for DNA extraction. DNA was finally eluted with 50 μl elution buffer supplied with Mo Bio soil DNA extraction kit. The concentrations of DNA from the two samples were determined by Nanodrop 2000 (Thermo Scientific, MA, USA).

The universal primer set 515F (5′-GTG CCA GCM GCC GCG GTA A-3′) and 806R (5′-GGA CTA CNN GGG TAT CTA AT-3′) was used for the amplification of the V4 region of bacterial 16S rRNA gene. The universal primer set U519F (5′-CAG YMG CCR CGG KAA HAC C-3′) and 806R (5′-GGA CTA CNN GGG TAT CTA AT-3′) was used for the amplification of the V4 region of archaeal 16S rRNA gene. All PCR reactions were carried out in 30 μl reactions with 15 μl of Phusion^®^ High-Fidelity PCR Master Mix (New England Biolabs, MA, USA), 0.2 μM of forward and reverse primers, and about 10 ng DNA. Thermal cycling consisted of initial denaturation at 98 °C for 1 min, followed by 30 cycles of denaturation at 98 °C for 10 s, annealing at 50 °C for 30 s, and elongation at 72 °C for 60 s, finally 72 °C for 5 min. The PCR products were analyzed on 2% agarose gel, and the DNA (~300 bp) was purified with GeneJET Gel Extrac-tion Kit (Thermo Scientific).

### 16S rDNA library preparation and sequencing

Sequencing libraries were generated using NEB Next^®^ Ultra™ DNA Library Prep Kit for Illumina (New England Biolabs) following manufacturer’s recommendations and different multipex indexing barcodes were added. The library quality was assessed on the Qubit@ 2.0 Fluorometer (Thermo Scientific) and Agilent Bioanalyzer 2100 system. The libraries were sequenced on an Illumina MiSeq platform 2500 and 250 bp paired-end reads were generated at Novogene (Beijing, China). Complete data were submitted to the NCBI Short Read Archive database under accession no. SRP062532 for bacterial sequences and no. SRP063077 for archaeal sequences.

### Quality filtering, OTUs picking and annotation

Raw data obtained from the Illumina MiSeq sequencing platform must be processed because they contain some low quality data that will interfere with analysis results. Based on the sequences of barcodes, raw data were demerged and then used to merge paired-end reads of each sample by using FLASH software^53^, when at least some of the reads overlap the read generated from the opposite end of the same DNA fragment. Paired-end reads was assigned to each sample according to the unique barcode. Barcode sequences and PCR primer sequences were also removed. The resulting sequences were defined as raw tags. The merged raw tag was strictly filtered and developed into clean tags with high quality. By means of QIMME (quantitative insights into microbial ecology)^54^, these clean tags were further processed. The site of first base from consecutive data with low quality (the default threshold value is < = 3) will be truncated if the base number is up to the defined length (the default length is 3). The truncated tags were developed into tags group. Among tags, some tags that contain base length with consecutively high quality is shorter 75% of tags length were further filtered. After processing mentioned as above, the obtained tags were blasted with Unite Database to detect chimera sequence by UCHIME Algorithm^55^. The chimera sequence was finally removed, and the effective tags were generated^56^.

During OTUs construction, the data of effective tags and tags with low quality, and tags annotated were analyzed. Effective tags with ≥97% similarity were clustered by Uparse^57^ and were classified into one OTU. For species analysis, sequences with ≥97% similarity were assigned to the same OTUs using Uparse, and similarity hits below 97% were not considered for classification purpose. A representative sequence of each OTU was picked out and the taxonomic information was annotated using RDP classifier^58^ and GreenGene database^59^.

### Diversity analysis

QIIME software package was used to analyze alpha (within samples) and beta (among samples) diversity. In alpha diversity analysis, the OTU tables were rarified and three metrics were calculated: Chao1 estimates the richness of microbial species; Observed Species estimates the amount of OTUs in each sample, and Shannon index estimates the diversity of microbial species. Based on these three metrics, rarefaction curves were generated. In beta diversity analysis, QIIME software package was also used to calculate both weighted and unweighted unifrac distance, which generates the beta diversity index.

## Additional Information

**How to cite this article**: Zhang, L. *et al.* Bacterial and archaeal communities in the deep-sea sediments of inactive hydrothermal vents in the Southwest India Ridge. *Sci. Rep.* **6**, 25982; doi: 10.1038/srep25982 (2016).

## Figures and Tables

**Table 1:** Geophysical features of sampling sites, mineralogical and geochemical characteristics of the samples.

Characteristic	TVG4	TVG11
Location	E 50.9277°, S 37.6251°	E 50.9643°, S 37.6174°
Sampling date	11 January 2009	14 January 2009
Depth (m)	2086	1985
Description	White sticky mud	Black loose sand
REE concn (mg/kg)^1^	19.82	21.08
Trace element concn (mg/kg)^2^		
Li	2.93	2.70
Be	0.06	0.06
Sc	1.54	1.27
V	8.55	8.23
Cr	6.38	5.53
Co	11.98	16.30
Ni	11.13	12.97
Cu	12.46	11.77
Zn	18.85	20.06
Ga	0.94	0.96
Rb	4.29	4.28
Sr	1494.00	1529.60
Zr	6.96	6.60
Nb	0.70	0.69
Cs	0.23	0.23
Ba	189.18	328.80
Hf	0.15	0.14
Ta	0.04	0.05
Pb	3.77	4.39
Th	0.41	0.48
U	0.18	0.17
X-ray diffraction phase analysis content (%)^3^		
Calcite	95.3	96.8
Quartz	0.7	0.4
Aragonite	1.3	
Clay minerals	2.7	2.8
Carbon and oxygen isotope analysis (%)^4^		
δ^13^C_PDB_	0.10	0.23
δ^18^O_PDB_	0.52	1.82

^1,2,3 and 4^Data are cited from Chen *et al.*^22^. REE: Rare Earth Element, PDB: Pee Dee Belemnite.

**Table 2:** Sequencing information in this study.

Sequencing information	Bacteria		Archaea	
	TVG4	TVG11	TVG4	TVG11
Number of total tags	45,414	33,047	180,951	177,638
Number of effective tags	43,139	31,841	148,922	162,113
OTUs (97% similarity)	366	280	19	19
Shannon index	5.76	3.99	2.05	3.05
Chao 1 index	371	283	19	19

**Table 3:** Potential ecological roles of the microbial organisms.

Potential role	Taxon	TVG4 (%)	TVG11 (%)
N cycling	*Nitrospira*	0.007	0.003
	*Rhizobiales*	1.3	0.9
	*Nitrosopumilus*	0.2	27.9
Metal oxidation	*Hyphomicrobium*	0.06	0.27
	*Rhodococcus*	0.02	0.003
	*Acinetobacter*	0.03	0.1
	*Caulobacter*	0.09	0.02
S oxidation	*Rhodobacteraceae*	6.88	4.09
	*Desulfobacterales*	0.007	ND
CH4 oxidation	*Planctomycetes*	0.005	0.003

ND: not detectable.
